# Stress Hyperglycemia as Predictive Factor of Recurrence in Children with Febrile Seizures

**DOI:** 10.3390/brainsci10030131

**Published:** 2020-02-27

**Authors:** Raluca Maria Costea, Ionela Maniu, Luminita Dobrota, Bogdan Neamtu

**Affiliations:** 1 Research and Telemedicine Center for Neurological Diseases in Children, Pediatric Clinical Hospital Sibiu, 550166 Sibiu, Romania; ionela.maniu@ulbsibiu.ro (I.M.); bogdan.neamtu@ulbsibiu.ro (B.N.); 2 Faculty of Medicine, Lucian Blaga University, 550024 Sibiu, Romania; luminitadobrota@yahoo.com; 3 Department of Mathematics and Computer Science, Faculty of Science, Lucian Blaga University, 550012 Sibiu, Romania; 4 Computer and Electrical Engineering Department, Faculty of Engineering, Lucian Blaga University Sibiu, 550025 Sibiu, Romania

**Keywords:** stress hyperglycemia, hyperlactatemia, febrile seizure, recurrence, metabolic status

## Abstract

Stress hyperglycemia and hyperlactatemia are commonly referred to as markers of stress severity and poor outcome in children with severe acute illness or febrile seizures. Our prospective study aimed to explore the risk factors for stress hyperglycemia and the predictive value of stress hyperglycemia for febrile seizure recurrence. We evaluated as risk factors for blood glucose level, serum lactate, acid–base status, and the clinical parameters relevant to the severity of the infectious context or to febrile seizure event: fever degree, fever duration, seizure type and aspect, seizure duration, and recurrence. Among 166 febrile seizures events in 128 children, the prevalence of stress hyperglycemia (blood glucose >140 mg/dl) was 16.9%. The comparison of the stress versus non-stress hyperglycemia groups revealed lower pH (median (interquartile range): 7.46 (7.37, 7.53) vs. 7.48 (7.42, 7.53), *p* = 0.049), higher lactate levels (30.50 mg/dl (15, 36) vs. 19.50 mg/dl (15, 27), *p* = 0.000), slightly lower HCO_3_ (20.15 (20.20, 21.45) vs. 21.35 (20, 22.40), *p* = 0.020) in the stress hyperglycemia group. Multiple logistic regression analysis showed that prolonged febrile seizures (>15 min), recurrent febrile seizure (>1 seizure), focal seizure type, body temperature ≥39.5 °C and higher lactate values were significantly associated with stress hyperglycemia. These findings suggest a particular acute stress reaction in febrile seizures, with stress hyperglycemia playing an important role, particularly in patients with a recurrent seizure pattern. A more complex future approach linking pathogenic mechanisms and genetic traits would be advised and could provide further clues regarding recurrence pattern and individualized treatment.

## 1. Introduction

Febrile seizures are reported among stress-related conditions correlated with stress hyperglycemia. Stress hyperglycemia is defined as transient high blood glucose levels, with spontaneous resolution after the acute illness regresses [1]. According to the latest American Diabetes Association and American Association of Clinical Endocrinologists consensus, the definition on stress hyperglycemia implies any transient inpatient plasma glucose levels > 140 mg/dl (fasting plasma glucose of >126 mg/dl or random plasma glucose > 200 mg/dl) without evidence of previous diabetes [2].

**Figure A1:**
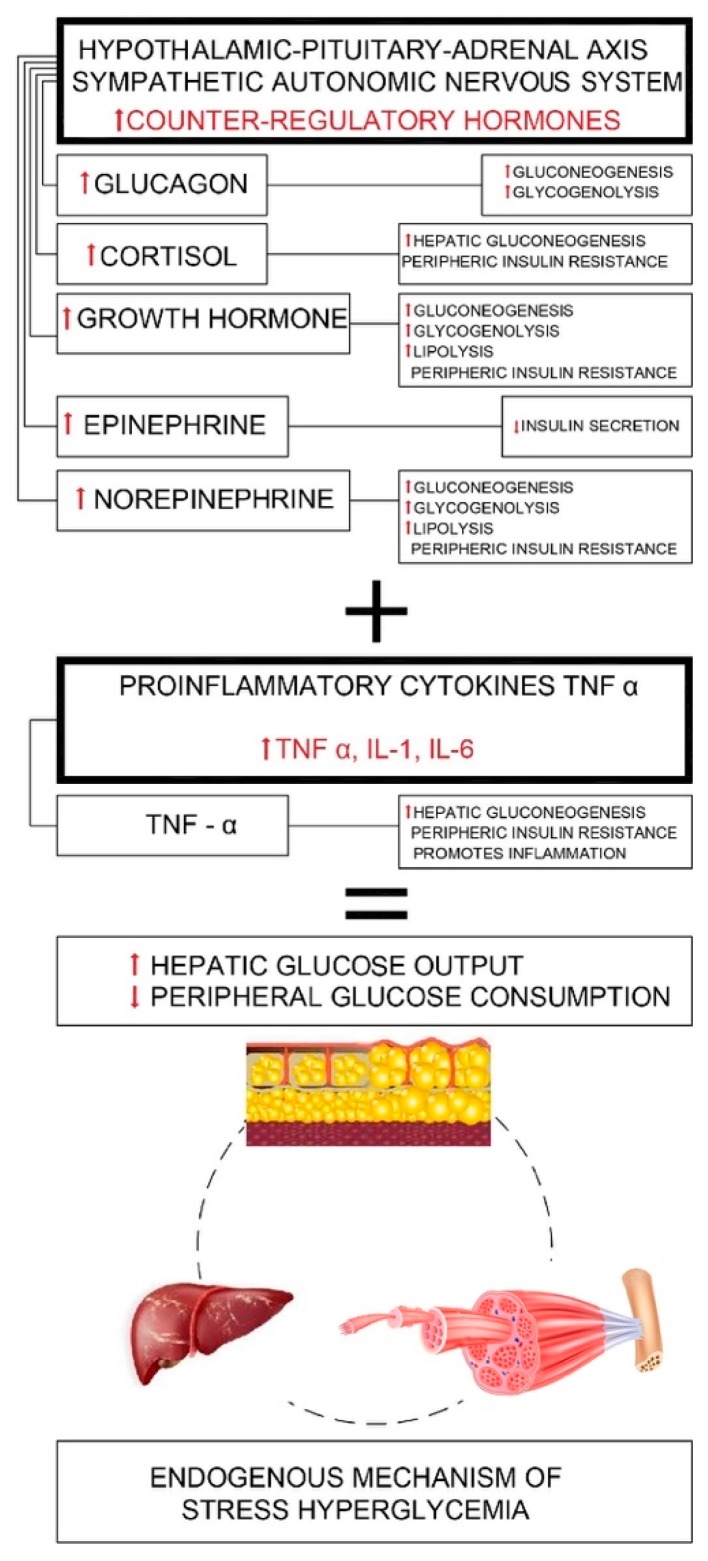
Endogenous mechanism of stress hyperglycemia.

The proposed triggering mechanisms in febrile seizures are temperature-sensitive ion channels and increased neuronal excitability in the context of proinflammatory cytokines [3]. Both seizure activity and fever-illness context have common stress-related mechanisms with high metabolic states, hypoperfusion, relative hypoxic states due to rising oxygen demands needed for additional energy supply, and accelerated glycolysis [1,4,5,6,7,8,9,10,11]. The oxidative stress is playing a key role as a common pathway for febrile seizures and stress hyperglycemia [8,12,13]. Moreover, in stress hyperglycemia, cytokines and hormones, interact in a complex manner supporting gluconeogenesis, glycogenolysis, and insulin resistance as pathogenic hallmarks [1,4,5,6,7,8,9,10,11]. Further details on the endogenous mechanism of stress hyperglycemia are available in Figure A1 and the text in the Appendix A. Currently, there are two physio-pathological approaches suggested, protective and/or complication-related, which describe the role of stress hyperglycemia in the prognosis of acute severe or chronic stress related conditions (trauma, severe infections, surgery, cardiovascular, cerebrovascular events). In febrile seizures, the intricate interplay of the neuroendocrine (catecholamines) and immune system pathways are common grounds for stress hyperglycemia and hyperlactatemia (Appendix A) [1,4,11]. Most research reports suggest that stress hyperglycemia and hyperlactatemia are more than markers of stress severity and participate in the complex adaptive and protective stress-related mechanisms supporting the survival response of the host to stress [1,4,5,11,14,15,16,17,18]. During stress, proinflammatory cytokines modulate glycemia levels upregulating GLUT-1 and downregulating GLUT-4 (plasma membrane glucose transporters) improve the redistribution of glucose toward the central nervous system, macrophage rich tissues and immune system [5], and cellular glucose uptake [5,18]. More than a byproduct of anaerobiosis, the lactate emerges as an important gluconeogenetic precursor, energy source, and as a key player for adapting to stress-related conditions [19,20]). It seems that high levels of lactate might have a dual protective role during the initial stage and at the end of the seizure event in the context of metabolic acidosis [21]. Further details on the mechanism of neuroendocrine responses, stress hyperglycemia, hyperlactatemia, and the immune system responses in febrile seizures are available in Appendix A. According to some other studies however, acute and long-term stress-induced hyperglycemia is currently believed to support a vicious cycle of self-promoting, exacerbating cytokine, inflammatory, and oxidative stress response [1,4,5,6,7,8,9,10,11]. Moreover, during febrile seizures, hyperlactatemia with or without acidosis reflects a complex metabolic disturbance, an imbalance between anaerobic and aerobic lactate production and clearance between the glycolytic and Krebs cycle activity either from the overproduction of pyruvate by increased glycolysis or from the underutilization of pyruvate, or from both [22].

In light of these reports, we aimed to explore, in a pilot study, the risk factors for stress hyperglycemia and stress hyperglycemia as a potential sensitive biomarker related to febrile seizure recurrence. Our main goal was to offer the practitioners a fast and easily accessible tool to assess the recurrence risk in febrile seizures. 

We evaluated the possible association of blood glucose levels with serum lactate, acid–base status, clinical parameters that define the severity of both infectious context, and febrile seizures (fever degree, fever duration, seizure type and aspect, seizure duration, and recurrence). 

## 2. Material and Methods

### 2.1. Design and Subjects 

We conducted a prospective study in the Pediatric Clinical Hospital from Sibiu, Romania between October 2013 and October 2016, on 128 children admitted with febrile seizures in the Emergency Department (ED). Parents of all eligible children gave their informed consent for inclusion before they participated in the study. The study was conducted in accordance with the Declaration of Helsinki and followed the principles of good clinical practice. The protocol was approved by the Ethics Committee of our institution. All the children presenting a characteristic and unequivocal history of a recent FS (febrile seizure) were included. The patients with central nervous system infections, other possible traumatic and metabolic causes of symptomatic seizures (dyselectrolytemia, hypoglycemia), preceding afebrile seizures, a pre-existing history of diabetes, uncertain or incomplete clinical data were not considered for enrolment. For children with recurrent febrile seizures, each seizure was treated as a distinct event, resulting in 166 febrile seizure events. We defined the cohort by applying the age criterion according to the revised ILAE (International League of Epilepsy) definition, namely age ranging between one month to five years [23]. A febrile context was documented if the temperature was higher than 37.5 °C prior to seizure event (up to 4 h) or immediately following the seizure event [24]. The simple febrile seizure was defined as a primary generalized seizure, lasting under 15 min and not recurring within 24 h. Complex febrile seizures were categorized as focal or prolonged seizures (>15 min) or seizures’ recurrence within 24 h [23]. The follow up period was 36 months.

### 2.2. Blood Glucose, Lactate and Acid–Base Status Evaluation 

First peripheral venous blood samples were taken upon arrival in the emergency department, on average within a 30-min time interval following the seizure, before any corticosteroid, beta adrenergic, and/or intravenous fluid administration. The follow up venous blood samples were taken up to 2 h after the seizure event. Blood glucose, lactate, and parameters defining acid–base status (pH, pCO_2_, HCO_3_) were documented for all venous blood samples. Current approaches envisage a cautionary view in evaluating the peripheral venous lactate instead of arterial lactate, especially for significant high value levels [25,26,27,28,29,30,31]. However, recent reviews on lactate measurement (arterial versus venous blood sampling) revealed very strong correlations between arterial and venous lactate concentrations [32]. Moreover, venous blood gas sampling is also currently emerging as an alternative method for the acid–base status assessment (pH, HCO_3_, pCO_2_ levels). A 2014 meta-analysis concluded a good pH and HCO_3_, but a less tighter pCO_2_ correlation between venous and arterial blood [33]. Although arterial blood sampling still represents the “gold standard", considering these recent data, we selected peripheral venous blood samples to be harvested. From a clinical practice perspective, it is acknowledged to be a more convenient method [34]. 

We interpreted the laboratory data for peripheral venous blood gas referring to pH within the normal range of 7.31–7.41; pCO_2_ 41–51 mmHg; HCO_3_ 24–27 mmHg, according to recent meta-analysis reinforcements [26]. Hyperlactatemia was defined as a mild to moderate increase of plasma lactate (2–4 mmol/L) without metabolic acidosis, occurring also in the context of adequate tissue perfusion and oxygenation and buffering systems [35,36,37,38]. Lactic acidosis implied elevated plasma lactate level (>4–5 mmol/L) associated with (high anion gap) metabolic acidosis [15,16,17,18]. Normal blood glucose level reported by our laboratory was in the range of 70–105 mg/dl.

### 2.3. Data Analysis

#### 2.3.1. Clinical and Laboratory Data

According to our study design, the enrolled patients were evaluated with a focus on glycemic status, general patient characteristics, seizure activity, and acute illness characteristics. Nutritional status was assessed using Word Health Organization growth charts (weight for length referred to gender under two years of age and BMI for age and gender over two years of age) [39]. We considered as potential predictors for glycemic status the variables age, gender, the body temperature at seizure onset, time interval between last fever episode and seizure onset, seizure duration, seizure types (simple or complex febrile seizures), seizure semiology (generalized or focal, with motor or non-motor characteristics), seizure recurrence, and multiple seizures within 24 h. Seizure duration was defined in four categories as less than 1 min, 1–4.9 min, 5–14.9 min, and longer than 15 min to study the distribution of cases related to these categories and in further two large categories: less than 15 min and longer than 15 min for the glycemic status analysis [40]. Time intervals from fever onset up to the seizure event and fever onset to fever remission were used as parameters for evaluating illness severity. The studied parameters were analyzed considering two main categories, namely stress hyperglycemia (blood glucose higher than 140 mg/dl) versus non -stress hyperglycemia (blood glucose levels lower than 140 mg/dl). A detailed analysis was also conducted between all parameters and three glycemia categories: normoglycemia (70–105 mg/dl), an intermediate hyperglycemia category (106–139 mg/dl), and stress hyperglycemia (over 140 mg/dl). The results were mainly presented for the two blood glucose levels over and under 140 mg/dl [40]. 

#### 2.3.2. Statistical Methods

The χ^2^-test or Fisher exact test for categorical data and T-test or Mann–Whitney U-test for continuous variables were appropriately used to compare the variables between groups (stress vs. non–stress hyperglycemia). We computed the means, medians, and interquartile range for the two groups. Risk factor analysis for stress hyperglycemia was conducted using the multivariable logistic regression method. We included the variables from univariate analysis with a *p* ≤ 0.25 as level of significance (a relaxed value for the pre-selection step) in the model, and then used the backward procedure to predict the outcome [41]. All statistical analyses were performed using SPSS software 20.0 (SPSS Inc, Chicago, IL, USA).

## 3. Results

### 3.1. Study Group Characteristics

Data analysis on the 128 patients identified 166 distinct febrile seizures events and 44 (26.5%) recurrent seizures. The mean age was 23.10 ± 12.03 months with a gender distribution of 93 (56%) male cases. From a nutritional status perspective comparing the two subgroups, in the non-stress hyperglycemia group 81.2% patients had normal weight, 9.4% were underweight, 7.2% overweight and 2.2% obese, while in the stress hyperglycemia group 96.4% were normal weight patients, 3.6% overweight, and none underweight or obese.

According to the seizure semiology, the most common findings for febrile seizure events in the admitted patients were generalized (97.6%), motor/convulsive seizures (72.9%), with overall simple febrile seizure diagnosis (86.7%), and average seizure temperature of 39.25 ± 0.73. Seizure duration between 1 and 5 min prevailed (71.1%). Prolonged seizures (over 15 min, 3.6%) and short events (under 1 min, 9%) were rare. From 22 (13.3%) complex febrile seizure cases, four cases presented focal seizures (2.4%), prolonged feature lasting 15 min or longer were reported in 10 cases (6.02%) and recurrences within 24 h in 8 cases (4.8%). Almost 65% of febrile seizures were associated with a first febrile episode, shortly preceding the seizure (up to 15 min). Most febrile seizures (91.5%) occurred during the first 24 h of febrile illness. In 107 cases (64.5%), there was a short time interval (under 15 min) between the febrile episode and febrile seizure, whereas in two cases, fever followed immediately after the seizure. Temperature normalization occurred within the first 24 h in 51.8% febrile seizures cases, and within the first 72 h in 86.7%. Only five febrile seizures events were associated with prolonged fever (over 72 h).

There were 28 (16.9%) febrile seizure events associating stress hyperglycemia, with a median blood glucose level of 16,700 (152.00, 180.50). More than half of the febrile seizure events (56.9%) reported hyperglycemia in the 106–139 mg/dl interval, median blood glucose level 11,350 (101.00, 128.00), and only 25.9 % normoglycemia. There were two patients with mild hypoglycemia, but not in the range of acute symptomatic seizure so we considered them eligible for our study. Severe stress hyperglycemia (over 200 mg/dl) was identified exceptionally in three patients, with the highest reported value of 212 mg/dl. Most patients had a rapid decline of stress hyperglycemia values, with normoglycemia reported at the up to two hours follow up from the admittance. Only two patients had a slower, but consistent glucose level decrease, reaching normal range up to 4 h.

None of the patients from the intermediate and stress hyperglycemia groups was diagnosed with diabetes during the 36 months follow up. 

### 3.2. Univariate Analysis on Clinical and Laboratory Characteristics in Stress versus Non- Stress Hyperglycemia Groups

Among the 18 studied parameters, univariate analysis highlighted the variables significantly associated with stress hyperglycemia: febrile seizure type (simple/complex), seizure duration, pH, lactate, and HCO_3_ levels. 

The number of patients with stress hyperglycemia was significantly higher in the case of complex febrile seizure (32.1% vs. 9.4%, *p* = 0.001) and prolonged febrile seizures exceeding 15 min (14.3% vs. 1.4%, *p* = 0.001). Complex febrile seizures cases had a four times higher risk for stress hyperglycemia (*p* = 0.001, OR = 4.55, 95%CI: (1.714, 12.104)) compared to simple febrile seizures. On the same note, there was a higher recurrence tendency in the stress hyperglycemia group (39.3%) compared to the non-stress hyperglycemia group (23.9%, *p* = 0.093, OR = 2.05, 95%CI: (0.877, 4.833)). 

Furthermore, in the stress hyperglycemia group, there was a shorter time interval, less than six hours between fever onset and seizure event (71.4% versus 55.1%, *p* = 0.110). More than half of the febrile seizure events in the stress hyperglycemia group (53%) were associated with a body temperature higher than 39.5 °C.

**Figure A2:**
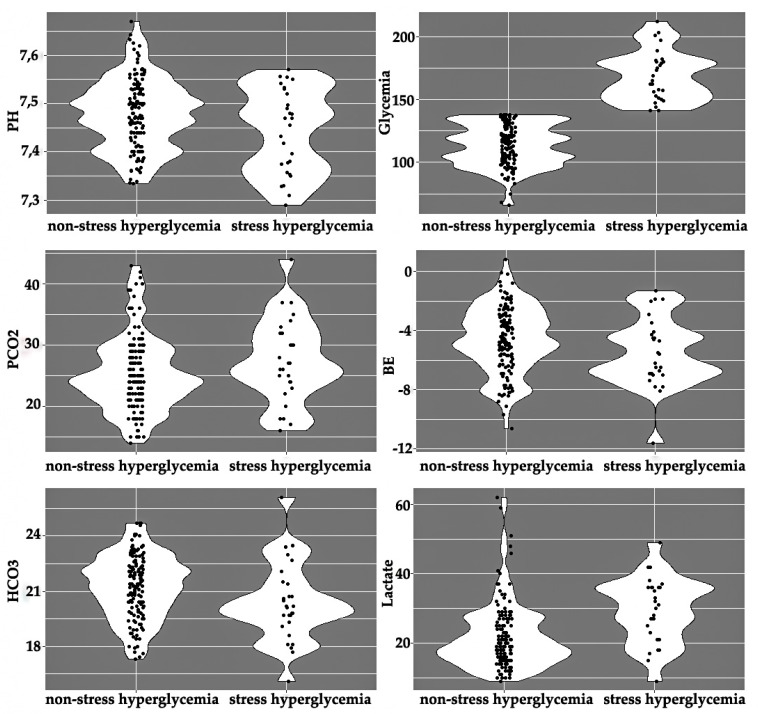
Acid–base status, glycemia, and lactic acid levels in the stress and non-stress hyperglycemia group (pH-logarithm of hydrogen ions concentration, glycemia-mg/dl, lactate-mg/dl, pCO_2_-mmHg, HCO_3_-mmol/L, BE-mmol/L).

The stress hyperglycemia group had lower pH (7.44 ± 0.08 vs. 7.47 ± 0.07, *p* = 0.049), slightly lower HCO_3_ (20.36 ± 2.10 vs. 21.23 ± 1.71 mmol/L, *p* = 0.020), and higher lactate levels (30.50 vs. 19.50 mg/dl, *p* = 0.000). Moreover, considering the three glycemic subcategories of normoglycemia vs. intermediate hyperglycemia vs. stress-hyperglycemia, we identified a direct association between blood lactate values and blood glucose levels (medians: 18 vs. 21 vs. 30.5 mg/dl) (see Appendix B, Figure A2).

We did not find any statistically significant difference between the groups for the other variables: seizure semiology (motor/non-motor), multiple seizures within 24 h, time interval between last fever episode, and seizure onset (Table 1). 

### 3.3. Multiple Logistic Regression Analysis in Stress Hyperglycemia Group

The multiple logistic regression method (with backward selection procedure) selected a 5-variable model to predict stress hyperglycemia, respectively prolonged febrile seizures (>15 min), focal seizure type, temperature ≥ 39.5 °C, recurrent seizures, and higher lactate values (>30 mg/dl) (Table 2). 

## 4. Discussion

We noticed a slightly higher prevalence of stress hyperglycemia in the febrile seizure cohort compared to the reports by Valerio et al. (12.9%) [42]. 

The fast remission of stress hyperglycemia did not impose insulin therapy in our study group, as mentioned recently by Fattorusso et al. [43]. Emergency Department standards of care included non-glucose hydroelectrolytic solutions and antipyretic treatment. None of the patients had persistent, 2 h severe stress hyperglycemia levels (over 200 mg/dl) to consider a more active, insulin targeted therapeutic approach. Although some pediatric studies involving patients with traumatic brain injury, sepsis, burns, surgical conditions have reported a better outcome in the group with strict glycemic control in comparison to the conventional approach, the results were inconsistent and were associated with a higher hypoglycemia risk. Currently, tight glycemic control using insulin therapy is controversial and the optimum timing of insulin therapy and target blood glucose uncertain, due to the conflicting results of the available literature data [43].

The main findings in our study pointed out that glycemic levels were significantly higher in children with complex febrile seizures, mainly in prolonged febrile seizures (over 15 min). Seizure duration was directly correlated with an increase in blood glucose levels, supporting prolonged febrile seizure as risk factors for stress hyperglycemia. The proposed mechanisms during prolonged seizures are related to aberrant, ongoing neuronal discharges leading to a high metabolic state, exacerbating aerobic glucose metabolism and anaerobic glycolysis [11]. The results are in accordance to Lee et al. and Chou et al.’s findings, reporting an association between hyperglycemia and prolonged seizure duration [40,44]. The univariate analysis could not establish any statistically significant association between the parameters defining fever/infectious context (fever duration, time interval between last fever episode, and seizure onset) and blood glucose levels. The time interval up to six hours between fever onset and seizure event was observed in a higher proportion in the stress hyperglycemia group, invalidating the value of prolonged infectious context as a risk factor for stress hyperglycemia. Although these findings suggest seizure activity as main risk factor for stress hyperglycemia, the exact contribution of seizure activity or febrile/infectious context to the development of stress hyperglycemia associated with febrile seizure remains uncertain. A complex interplay between fever, seizure, and infection as combined stressors, triggering a cumulative, synergic interaction between proinflammatory cytokines and stress-related hormones (interleukins, growth hormone, insulin, glucagon) could offer a potential explanation (Appendix A) [42]. 

Moreover, other studies have reported higher stress hyperglycemia prevalence in febrile seizures in contrast to seizure events not precipitated by fever or by fever without seizure events [42]. Valerio et al. reported in the presence of severe stress, defined in this study by the combined presence of fever and seizures, stress hyperglycemia with a three times higher frequency than in other febrile conditions (12.9 % vs. 4%) and a five times higher frequency than in seizures without fever (12.9% vs. 2.4%) [41]. In our study, pH, pCO_2_, and HCO_3_ levels suggested respiratory alkalosis in most febrile seizure events. The results are in agreement with other findings correlating febrile seizures with hypocapnia and respiratory alkalosis through fever induced hyperventilation [45,46]. Lower seizure threshold of the brain receptors associated with alkalosis, and brain alkalosis as promoters of epileptic activity and enhancers of neuronal excitability have been proposed as possible seizure mechanisms [47,48]. 

The study identified an important association with statistical significance between hyperlactatemia and stress hyperglycemia. A central tendency of 18 mg/dl in the normoglycemic group, 21 mg/dl in the intermediate hyperglycemia group, and 30.5 mg/dl in the stress hyperglycemia group revealed a direct increase of blood lactate with blood glucose. Our results are consistent with the current available literature data in febrile seizures. A complex interplay of the neuroendocrine (catecholamine) and immune system seems to be the common ground for stress hyperglycemia and hyperlactatemia. In this context, high lactate levels might have a dual protective role in the initial stage and at the end of the seizure events in the context of metabolic acidosis. A transitory high metabolic demand, hypoperfusion, a relative hypoxic state due to rising oxygen demand needed for additional energy supply, and accelerated glycolysis are common stress related mechanisms for the fever-illness context and seizure activity [19,20]. Hyperlactatemia is acknowledged as the result of dysbalance between lactate production and lactate uptake and clearance mainly through the Cori cycle. In febrile seizures, two mechanisms determine an increase in lactate production namely accelerated aerobe glycolysis by catecholamine and anaerobe glycolysis by partial local muscle tissue hypoxia during massive increase in muscular activity.

The systemic alkalosis demonstrated in our febrile seizure study group could explain the impaired lactate liver uptake with subsequent decreased lactate clearance [49,50,51]. Another known mechanism is stimulation of phosphofructokinase during alkalosis, promoting glycolysis and lactate production [52]. 

Furthermore, our study demonstrated a two times higher recurrence risk tendency in the stress hyperglycemia group compared to the non-stress hyperglycemia group. In contrast to our results, Lee et al. described the children with first-time febrile seizure as prone to stress hyperglycemia, arguing possible higher thresholds for the initiation of neuronal changes in first-time febrile seizures than in recurrent febrile seizure [40]. Although peak and persistent high blood glucose levels are independently associated with poor outcome in the pediatric Intensive Care Unit current research is limited in providing a consistent cause–effect relationship between stress hyperglycemia and prognosis. Most studies targeting the harmful impact of hyperglycemia include persistent high glucose concentrations as promoters of cellular cytotoxic effect through mitochondrial damage protein and exacerbated inflammatory pathways. More recent studies, although scarce, suggest the potential, “toxic” effects of chronic but also acute hyperglycemia in modifying the innate immune system, impairing endothelial function, reducing vascular reactivity, and endothelial nitric oxide production, which may compromise peripheral blood flow. In addition, hyperglycemia can alter polymorphonuclear neutrophil function, opsonic, and bactericidal activity promoting infections. Acute hyperglycemia may enhance proteolysis, cardiac, and renal complications. Moreover, the benefit of moderate glycemic control in patients with severe hyperglycemia (over 220 mg/dl) indirectly supports its harmful potential [43]. In our study, despite the association of stress hyperglycemia with higher febrile seizure recurrence, there was a rapid, consistent decline of stress hyperglycemia values up to two hours after admittance. Only a few patients experienced severe stress hyperglycemia (three values over 200 mg/dl, highest glucose level 212 mg/dl), consequently, we cannot advance glucotoxicity and stress hyperglycemia as a promoter of febrile seizure recurrence [43].

Our results are in agreement with the current research addressing stress hyperglycemia and prognosis, in our case febrile seizure recurrence, from the perspective of associated factors and not as a cause and effect relationship. Nevertheless, the results support the practical value of stress hyperglycemia as a predictive tool or prognosis indicator for febrile seizure recurrence. Current research supports an association between repeated febrile convulsions and the risk of epilepsy and psychiatric disorders. A population study showed a risk of epilepsy of approximately 15% within thirty years among children who had three or more recurrent febrile seizures. The risk of a psychiatric disorder requiring treatment was high, approximately 30%. In comparison, in children without prior febrile convulsions, the risk of epilepsy was approximately 2% and of developing a psychiatric disorder was 17% [53,54].

At a glance, our study did not aim for an exhaustive assessment of the pathophysiological mechanisms involved in stress hyperglycemia or febrile seizures. We evaluated biomarkers that are always available in clinical practice in order to easily assess the risk of recurrence in febrile seizures. To the best of our knowledge, the literature is scarce in this respect. Despite the restricted number of patients in the stress hyperglycemia group, the statically significant association between prolonged febrile seizure, febrile seizure recurrence, and stress hyperglycemia should not be ignored, but further explored.

There are some limitations regarding our study. It is a single center experience, although similar study results focusing on similar biomarkers sets are available. Prospective, multicentric, and larger sample studies are necessary strengthen our findings. Body temperature and fever duration provide insufficient data about the infectious/febrile status. Records about seizure duration rely on the subjective assessment of anxious parents due to the dramatic nature of the seizure event and could partially influence statistical analysis on tight seizure duration categories. Data analysis on course seizure duration categories (under and over 15 min) is, however, eloquent. To validate higher focal seizure presence in the hyperglycemia group, a larger number of focal febrile seizures is necessary. 

Our study did not identify risk factors associated with low glucose tolerance as no patients with a first degree family history of diabetes were identified in the stress hyperglycemia group. Furthermore, obesity, as an insulin resistance associated condition, did not prevail in the stress hyperglycemia group in comparison to the non-stress hyperglycemia group. During the follow up, none of the patients with stress hyperglycemia developed symptoms of diabetes, although previous studies indicated a high risk time interval for diabetes occurrence within 12–18 months after transient hyperglycemia in a non-severe illness [42]. Nevertheless, assessing low glucose tolerance (HbA1c, peptide C, insulin) early, during the acute condition associated with stress hyperglycemia, and in dynamics could provide more data about low tolerance glucose status. 

As a final remark, we consider that further specific laboratory parameters in defining the severity of the stress context (insulin, prolactin, glucagon, cortisol, growth hormone, interleukins) and preexisting glucose homeostasis disturbances could provide more insights and are proposed for future research. The analysis of pyruvate levels and urine lactate levels in relation to blood lactate levels could offer an interesting perspective, adding more data upon metabolic status in febrile seizures. Moreover, we believe that the analysis of the relationship between stress hyperglycemia and certain genetic traits as background for the individual reactivity to the febrile seizure context could also be the object of further research approaches. In this context, identifying new biomarkers as predictors for febrile seizure recurrence could support individualized treatment and follow-up, adding more possible predictors for febrile seizure long-term outcome.

## 5. Conclusions

The prevalence of stress hyperglycemia is relatively high in children with febrile seizures. Children with complex febrile seizures and especially prolonged febrile seizures (exceeding 15 min) are prone to stress hyperglycemia. Our research identified a significant statistical association between hyperlactatemia and stress hyperglycemia, supporting anaerobic glucose metabolism in febrile seizures. The higher percentage of febrile seizure recurrence in the stress hyperglycemia group suggests stress hyperglycemia as a fast, accessible tool in assessing the recurrence risk of febrile seizures. Multicentric and larger sample studies linking genetic pathology with febrile seizure prognosis would provide more insights into stress hyperglycemia as a predictor of seizure recurrence.

## Figures and Tables

**Table 1:** Comparison of characteristics for children with stress hyperglycemia versus non-stress hyperglycemia group.

	Non-Stress Hyperglycemia Group <140 (*n* = 138)	Stress-Hyperglycemia Group ≥140 (*n* = 28)	Sig.
Age (months) ^a^	23.38 ± 12.22	21.75 ± 11.15	0.516
Gender (m/f)	71 (51.4%)/67 (48.6%)	22 (78.6%)/6 (21.4%)	0.008
Body temperature (°C) ^a^	39.24 ± 0.70	39.34 ± 0.88	0.489
Body temperature (≥39.5 °C) ^b^	56 (40.6%)	15 (53.6%)	0.205
Seizure semiology (generalized/focal) ^b^	136 (98.6%)/2 (1.4%)	26 (92.9%)/2 (7.1%)	0.073
Type of seizure ( simple/complex) ^b^	125 (90.6%)/16 (9.4%)	19 (67.9%)/9 (32.1%)	0.001
Seizure semiology (motor/non motor) ^b^	99 (71.7%)/39 (28.3%)	22 (78.6%)/6 (21.4%)	0.458
Time interval from fever onset to seizure event (<6 h/≥6 h) ^b^	76 (55.1%)/62 (44.9%)	20 (71.4%)/8 (28.6%)	0.110
Time interval from last fever episode seizure (<15 min/<1 h/1–6 h/6–24 h) ^b^	91 (65.9%)/21 (15.2%)/ 14 (10.1%)/ 12(8.7%)	18 (64.3%)/3 (10.7%)/ 3 (10.7%)/ 4(14.3%)	0.778
Recurrence of FS >1 episode	33 (23.9%)	11 (39.3%)	0.093
Multiple FS within 24 h	7 (5.1%)	1 (3.6%)	0.597
Seizure duration (≤15/>15 min)	136 (98.6%)/2 (1.4%)	24 (85.7%)/4 (14.3%)	0.001
pH ^a^	7.47 ± 0.07; 7.48 (7.42, 7.53)	7.44 ± 0.08; 7.46 (7.37, 7.53)	0.049
Anion-gap ^a^	3.88 ± 3.29; 4.25 (1.90, 5.70)	4.64 ± 3.00; 5.05 (1.55, 6.05)	0.264
Lactate (mg/dl) ^a^	19.50 (15.00, 27.00)	30.50 (15.00, 36.00)	0.000
Glycemia (mg/dl) ^a^	113.50 (101.00, 128.00)	167.00 (152.00, 180.50)	0.000
PCO_2_ (mmHg) ^a^	24.35 (21.10, 28.70)	26.55 (21.25, 32.15)	0.074
BE (mmol/L) ^a^	−4.62 ± 2.22; −4.60 (−6.00, −2.90)	−5.49 ± 2.31; −5.90 (−6.95, −4.20)	0.061
HCO_3_ (mmol/L) ^a^	21.23 ± 1.71; 21.35 (20.00, 22.40)	20.36 ± 2.10; 20.15 (20.20, 21.45)	0.020

^a^ mean, standard deviation+/− IQR, ^b^ number of cases and percentages; *p*-values were computed using χ^2^-square or Fisher exact test for categorical variables and T test or Mann–Whitney U-test for continuous variables.

**Table 2:** Multiple logistic regression model to predict stress hyperglycemia.

Variable	OR (95% CI)	*p*-Value
Lactate (>30 mg/dl)	1.098 (1.047, 1.151)	0.000
Recurrence of FS > 1 episode	3.673 (1.285, 10.499)	0.015
Type of seizure (focal)	16.993 (1.217, 237.186)	0.035
Body temperature (≥39.5 °C)	2.875 (1.025, 8.061)	0.045
Seizure duration (>15 min)	20.852 (2.356, 184.568)	0.006

## Appendix A

Figure A1 provides a structured overview of the endogenous mechanism of stress hyperglycemia. Stress hyperglycemia is characterized by a complex interplay between the proinflammatory cytokines (immune system) and hypothalamic–pituitary–adrenal axis, sympathetic autonomic nervous system with counter-regulatory hormone release (growth hormone, catecholamine, and cortisol) as neuroendocrine mechanisms. The results are excessive gluconeogenesis, insulin resistance, and stress hyperglycemia [1,4,5,6,7,8,9,10,11].

Insulin resistance is mainly characterized by reduced insulin-mediated glucose uptake secondary to defects in postreceptor insulin signaling and altered glucose transporter. Secondary diminished muscle glycogen synthesis impairs the non-oxidative glucose disposal. Insulin resistance ultimately induces lipolysis, which promotes an ongoing vicious cycle by exacerbating insulin resistance through altering end-organ insulin signaling and glycogenogenesis. Acute illness is characterized by a global insulin-resistance syndrome secondary to the toxic effect of glucose through oxidative stress, fatty acids, and inflammation [1,4,5,6,7,8,9,10,11]. During acute severe illness, the role of proinflammatory cytokines in inducing stress hyperglycemia is complex. The role of inflammatory cytokines in the pathogenic cascade of febrile seizures is supported by several studies and could provide the common pathways with stress hyperglycemia [55,56,57,58]. Proinflammatory cytokines like TNFα, interleukin 1, interleukin 6, and interleukin-8 promote insulin resistance by inhibiting postreceptor signaling and activate the hypothalamic–pituitary–adrenal axis increasing the glucocorticosteroid and cathecolamine secretion. TNFα stimulates lipolysis by enhancing the expression of hormone-sensitive lipase. It stimulates glucagon production and facilitates gluconeogenesis. Interleukin-1 increases the secretion of corticosterone and glucagon associated with glucose production and by activating the inhibitor-kB kinase in the hepatocytes, it is correlated with the gene expression of inflammatory cytokines, finally suppressing GLUT4 translocation. Glucose transporter translocation is essential for cell glucose uptake [1,4,5,6,7,8,9,10,11].

Furthermore, exploring the seizure mechanisms reported by Yang et al., there is common ground regarding hyperlactatemia and stress hyperglycemia during febrile seizure as follows. In febrile seizure, the normal synchronized discharge of neurons leads to a high metabolic state, high oxygen and energy demand, promoting aerobic glucose metabolism and anaerobic glycolysis. The relative brain hypoxic state alters aerobic metabolism, promoting the glycolytic pathway through the increase in the transcriptional level of the glycolytic metabolic molecule hypoxia inducible factor secondary to the inhibition of key enzyme activity involved in the Krebs cycle and mitochondrial oxidative stress. Brain glycogenolysis in astrocytes offers the substrate for lactate production, with energy supply provided through the astrocyte–neuron lactate shuttle pathway and by the gap junction-mediated metabolic cycle pathway. Acting as an important source of biofuel, lactate has a protective role during the initial stage of the seizure [21]. The potential role in seizure termination relies on metabolic acidosis and energy consumption [21,59,60,61,62]. Acidemia as a consequence of elevated lactate levels is dependent on the severity of hyperlactatemia, the buffering systems’ efficiency (bicarbonate, hemoglobin, proteins), and precipitating conditions associated with tachypnea and subsequent alkalosis [35,36]. Lactate suppresses the voltage-gated Na^+^ and Ca^2+^ channel activity [21,63], inhibits the activity of a key glycolitic enzyme 6 diphosphate kinase-1 [21,64,65,66] and N-methyl-D-aspartate receptors [21,67], activates the acid-sensitive 1A ion channel, increasing the inhibitory γ-aminobutyric acid neurotransmitter [21,68,69] and strengthening the receptive mechanism of the inhibitory neurotransmitter γ-aminobutyric acid [21,63,64,65,66,67]. Kidneys account for up to 2% lactate disposal under physiological conditions and up to 25–30% under stress conditions with severe hyperlactatemia [25]. Therefore, urine lactate may be a novel biomarker of lactate production in the febrile seizure context, but its reliability depends on the laboratory analysis method and could raise some issues when assessed in the ICU unit [70,71].

After seizures, large amounts of energy are needed to re-establish ion gradients and membrane potentials as well as to repair damage [22]. Therefore, pyruvate assumes the critical role of starting point for gluconeogenesis and is critical for mitochondrial ATP generation and for driving several major biosynthetic pathways intersecting the citric acid cycle [22].

Although the mechanisms of febrile seizure are still under debate, the immune activity may overlap with the counter regulating hormonal activity and proinflammatory status described in stress hyperglycemia [1,4,5,72,73]. In febrile seizures, the role of inflammatory cytokines in the pathogenic cascade of febrile seizures has been supported by several studies [55,56,57,58].
